# Unintended pregnancy among female sex workers in Mekelle city, northern Ethiopia: a cross-sectional study

**DOI:** 10.1186/s12889-015-1366-5

**Published:** 2015-01-31

**Authors:** Rishan Weldegebreal, Yohannes Adama Melaku, Mussie Alemayehu, Tesfay Gebregzabher Gebrehiwot

**Affiliations:** College of Health Sciences, Mekelle University, Mekelle, Ethiopia; Department of Public Health, College of Health Sciences, Mekelle University, PO Box 1871, Mekelle, Ethiopia

**Keywords:** Female sex workers, Unintended pregnancy, Contraceptive, Ethiopia

## Abstract

**Background:**

Unintended pregnancy is a significant public health concern in the world. Particularly, female sex workers are exposed to the risk of unintended pregnancy, abortion and their consequences. The aim of this study was, therefore, to assess unintended pregnancy and associated factors among female sex workers in Mekelle city, northern Ethiopia.

**Methods:**

A community based cross-sectional study was conducted among 346 female sex workers from five *Kebelles* (smallest administrative units in Ethiopia) of Mekelle city from March-April, 2014. Sex workers were selected with simple random sampling technique using sampling frame obtained from urban health extension program. Epi-data version 3.1 was used to enter data and analysis was done using SPSS version 20. Bivariate and multivariate logistic regressions were performed to identify factors associated with unintended pregnancy using odds ratio and 95% confidence interval with P-value of 0.05.

**Results:**

The magnitude of unintended pregnancy among female sex workers in the past two years was 28.6%. During this period, 59 women had abortion which represents three-fifths, (59.6%), of those who had unintended pregnancies, and 17.1% of all female sex workers. Female sex workers who gave birth and had history of abortion formerly had 3.1 (AOR = 3.07, 95% CI: [1.54, 6.09]) and 15.6 (AOR = 15.64 95% CI: [8.03, 30.47]) times higher odds of unintended pregnancy compared to their counterparts, respectively. Sex workers who had steady partners had 2.9 (AOR = 2.87, 95% CI: [1.47, 5.61]) times higher odds of have unintended pregnancy than those who hadn’t. Drug users had 2.7 (AOR = 2.68, 95% CI: [1.30, 5.52]) times higher odds of unintended pregnancy than those who hadn’t use. Sex workers who had 60–96 months of duration in sex work were 67% less likely to have unintended pregnancy than those with <12 months (AOR = 0.33, 95% CI: [0.11, 0.95]).

**Conclusions:**

High level of unintended pregnancy and a range of associated factors were identified among sex workers. Improving utilization of effective pregnancy prevention methods in a consistent manner can avert the existing high level of unintended pregnancy among female sex workers.

**Electronic supplementary material:**

The online version of this article (doi:10.1186/s12889-015-1366-5) contains supplementary material, which is available to authorized users.

## Background

Unintended pregnancy is a pregnancy that is either unplanned or unwanted at the time of conception. It is significant public health concern in the world due to its negative association with social and health effects for both women and their families in particular and to health sector’s resources and the public at large [1,2].

Of the estimated 210 million pregnancies that happen throughout the world every year, about 40% are unintended and around 14 million are from sub-Saharan Africa [3]. The World Health Organization (WHO) estimates nearly 5.5 million African women practice unsafe abortions each year. Over 36,000 of these women die from the procedure, while millions more suffered from short or long term illness and disability. This case is not only in countries where abortion is permissible and secure but also in places where it is prohibited [3,4].

Globally, unintended pregnancies are major public health problems among key population such as youths and Female Sex Workers (FSWs) with high rate of sexual risk behavior [4,5]. In sub-Saharan Africa, women who are engaged in commercial sex are at high risk of physical and sexual violence, unwanted pregnancy, and Sexually Transmitted Infections (STIs) [6].

The prevalence of women with unintended pregnancy was reported as 24%, according to the Ethiopian Demography Health Survey (EDHS) 2011. This contributes to the deaths of many hundreds of women in the country which remains still with the highest ratio (676 deaths per 100,000 live births in the year 2011) [7,8]. Studies from different regions of Ethiopia revealed that the rate of unintended pregnancy varies in the range of 27.9% to 42.4% which is an evidence to be among the main causes of maternal mortality [9-11].

Despite the concern of many governments about the rising rates of unintended pregnancy and unsafe abortion, still access to safe abortion is restricted especially in low income countries where unsafe abortion causes more than 30% of maternal deaths [5,7]. To the best of our knowledge, very few studies have reported on unintended pregnancy among FSWs in sub-Saharan Africa. These studies have shown high incidences of unintended pregnancy and low contraceptive use in this particular group ([5,6] *Mamboleo N: Unwanted pregnancy and induced abortion among female youths: a case study of Temeke district, Dar es Salaam, Tanzania, unpublished*).

Usually, the primary focus of previous researches and interventions that have been conducted on FSWs emphasize on vulnerability to STIs, particularly Human Immunodeficiency Virus (HIV) infection. However, such targeted programmes sometimes overlook the broader reproductive health needs of these women. As a result, inadequate attention has been paid to the issues related to FSWs susceptibility and risks related to unintended pregnancy [6,12,13]. There might be high potential of other health related problems such as STIs, HIV and unsafe abortion following the unintended pregnancy which in turn could lead to high morbidity and mortality of FSWs. The society, in general, also could beat high risk of acquiring infections as FSWs are core transmitters of venereal diseases including HIV.

Therefore, this study has tried to fill the gap by emphasizing to identify associated factors of unintended pregnancy among FSWs. Stakeholders and concerned bodies could use findings of this study to design interventions among FSWs to protect unintended pregnancy and related consequences. Furthermore, to the best of our knowledge, there are no reports in relation to unintended pregnancy among FSWs in Ethiopia. Hence, the study will serve as an input for further investigations.

## Methods

### Study area

This study was conducted in selected sub-cities of *Mekelle* which is a capital of *Tigray* regional state and located 783 kilometer in the north from the capital city of Ethiopia, Addis Ababa. Administratively, Mekelle is divided into 7 sub-cities which are sub-divided to 33 *Kebelles* (the lowest administrative units in Ethiopia).

According to the Central Statistics Agency, the projected population size of Mekelle is 286,600 [14]. Mekelle is among the cities where construction boom and fastest urbanization is undergoing. There are 11 public health institutions (one referral, one general hospital and nine health centers) and 4 general hospitals and 38 clinics that are owned by private sectors. In addition, there are two non-profitable clinics called Family Guidance Association of Ethiopia (FGAE) and Confidential STI clinics. The FGAE clinic provides services focusing on reproductive health services to the general population, whereas confidential STI clinic which is run under the University of Mekelle emphasizes on delivering comprehensive promotive, preventive and curative services on STIs/HIV for most at risk group population especially to FSWs and their partners.

### Study design and data collection procedures

A community based cross-sectional study design was conducted in Mekelle city, Tigray region, Northern Ethiopia. Women who were working as FSWs at least 6 months prior to the data collection and who are capable of being interviewed in the selected Kebelles of the sub-cities were considered in the study.

Data were collected using structured questionnaire from March-April, 2014. The questionnaire included socio–demographic characteristics (age, religion, education, ethnicity, income, duration of stay in sex work, number of families) and behavioral and related variables (consistent condom use, number of sexual partners, drug use and having regular sexual partner), condom breakage and slip. The questionnaire was designed in English and translated to local language “Tigrigna”. Experienced persons with excellent knowledge in both languages translate the “Tigrigna” version back to English. Four urban health extension workers with qualification of diploma in nursing were involved in the data collection. In addition, two social workers with qualification of Bachelor degree have dealt in supervisory activities.

### Variables and operational definitions

The main outcome variable of the study was unintended pregnancy. Unintended pregnancy is defined as a pregnancy that is either unplanned or unwanted or both at the time of conception for the last 2 years [1]. Predictor variables including socio-demographic, behavioural and health care related characteristics, as well as obstetric factors were incorporated. In this study, abortion includes all type (safe, unsafe, induced and spontaneous) abortions. *Risky sexual practice* was defined as having sexual contact history with causal partners, multiple regular partners or experiencing unprotected sex (having sex without condom or without any method of pregnancy prevention) or all the stated definitions in the past two years. *Sexual contact* was defined as having penetrative penile-vaginal sexual intercourse. In this study, *drug use* was defined as taking psycho-active substances (like Khat, Hashish and others) at least once a day to be alert or to perform daily activities.

*Female Sex Workers were* defined as those who are trade sex workres for money in drinking establishments, night clubs, local drink houses, on the street and at their homes. *Condom breakage* was defined as breaking of a condom during sexual intercourse. *Condom slippage* is slipping-off of a condom from a penis completely during sexual intercourse. *Consistent condom use* is defined as using condom at every sexual intercourse. *Regular sexual partner-* was defined as spouse or co-habiting sexual partner. The *number of sexual partner* refers to the number of spouse or co-habiting sexual partner. Unless specified, all definition of the variables refers to a time which a woman started commercial sex work and within the past two years.

### Sample size and sampling procedure

The sample size was determined using single population proportion formula. The following assumptions were taken into consideration. The prevalence of unintended pregnancy among FSWs assumed to be 52% [13], marginal error of 5%, and at 95% level of confidence. Thus, the required sample size was calculated as 346. Since the number of FSWs population in the city is less than 10,000 (3500 in January 2014), the sample size was corrected and 10% of non-response rate was considered to give final minimum sample size of 315. However, data from 346 FSWs was collected.

**Figure 1:**
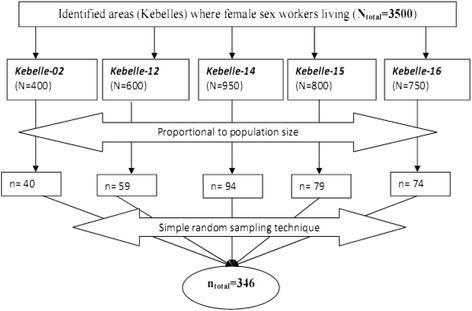
**Schematic representation of the sampling procedure.**

Fully documented data on the number, lists and addresses of FSWs in each kebelle of the sub-cities were found from urban health extension program of the municipal health office. Of the 33 kebelles, the residences of FSWs were identified in five Kebelles (Kebelles 02, 12, 14, 15 and 16). In each Kebelle, the number of study participants were determined using proportional allocation to population size. In addition, simple random sampling technique (using Microsoft Excel) was applied to select a study participant for interview in each Kebelle. Detail sampling techniques are depicted in Figure 1.

### Data management, quality control and analysis

Data collectors and supervisors were trained for three days on the purpose of the study, methodology, content of the study tool, interviewing approaches and data recording. Pre-test was performed on 5% of the FSWs who reside outside the selected Kebelles. A revision was made on some variables (like contraceptive use) of the questionnaire. Time needed per questionnaire was also determined after pre-testing.

The collected data were checked by supervisors and investigators on daily bases for completeness, consistency and accuracy. Codes were given for each questionnaire and entered using Epi-data version 3.1. Cleaning and analyses of data was performed using SPSS version 20 (*SPSS*, *IBM, New York*). Descriptive analysis was used to determine prevalence of the unintended pregnancy and other characteristics of the participants. Bivariate and multivariate logistic regressions with enter mode were used to identify potential predictors of unintended pregnancy. Variables that showed significant association with unintended pregnancy in the bivariate analysis at P-value <0.05 were entered into multivariate analysis. Odds ratio with 95% confidence interval were used to measure association between the predictors and the outcome variable. All variables in the multivariate model with P < 0.05 were considered as independent predictors.

### Ethical considerations

Information letters and written consent forms were available in English and “Tigrigna”. Ethical clearance was obtained from the Institutional Review Board of Mekelle University, College of Health Sciences. All concerned bodies were officially communicated through letters and permission was obtained at all levels, including the municipality health office. After explaining the purpose and contents of the study, oral consent was obtained from each respondent.

All the interviews with study participants were conducted in strict privacy, and confidentiality was assured. Each interview was conducted in a separate room. The participants were told that they have the right to either refuse to participate in the study or to withdraw after responding to some of the questions for any reasons without any consequences. Respondents’ names and identities were not recorded in the questionnaire in any way. Data were kept confidential by locking the completed questionnaires in boxes with a key, and by use of a password in the computers where data were stored so as to avoid access by any third party. The collected data contained no links to any individual identities at any stage of data processing and analysis.

## Result

### Socio-demographic characteristics

A total of 346 FSWs participated in the study and the response rate was 100%. More than one third, 135 (39.0%), of the respondents were between the age of 20 and 24 years with mean age of 22.6 (Standard Deviation ±4.98). One hundred ninety eight (57.3%) of the respondents had no regular partner. Almost half of them, 180 (52.0%), attended primary school. Nine out of ten (88.7%) FSWs were “Tigrian” in Ethnicity (Table 1).

**Table 1:** **Socio-demographic and economic characteristics of female sex workers in Mekelle city, northern Ethiopia, 2014 (n = 346)**

**Characteristics**	**Frequency (n)**	**Percent (%)**
**Age in years**		
15–19	109	31.5
20–24	135	39.0
25–29	64	18.5
>30	38	11.0
**Relationship status**		
Had no regular partner	198	57.3
Had regular partner	87	25.0
Dissolved (widowed and divorced)	61	17.7
**Educational status**		
Illiterate	62	17.9
Literate	284	82.1
**Grade fulfilled (n = 284)**		
Elementary (1–8)	180	52.3
High school (9–12)	81	23.4
Higher education	23	6.4
**Ethnicity**		
Tigrian	307	88.7
Others*	39	11.3
**Religion**		
Orthodox	339	97.7
Others^@^	7	2.3
**Place of work**		
Bar	79	22.8
Hotel	35	10.1
Night club	30	8.7
Open door (in-house)	167	48.3
Street, coffee house and others^#^	35	10.1
**Additional work**		
Yes	40	11.5
No	306	88.5
**Type of additional work (n = 40)**		
Micro and small activity	20	50.0
Daily labor	8	20.0
Waiter	6	15.0
Coffee house, housemaid	6	15.0
**Monthly income in USD**		
<=50	127	36.7
50–100	129	37.3
>100 and < =150	59	17.0
>150	31	9.0
**Birth place**		
Urban	174	50.3
Rural	172	49.7
**Arrived from out of Mekelle**		
Yes	293	84.7
No	53	15.3
**Reason leaving birth place (n = 293)**		
Searching for better life	167	57.0
Fear of early marriage, war or instability	31	10.6
Family collapse	67	22.9
Others^$^	28	9.5

*Amhara, Oromo; ^@^Muslim, protestant; ^#^telephone call; ^$^peer pressure, marriage.

One hundred sixty seven (48.2%) of the FSWs reported that their living house (indoor) is used as working place. The median income per month of FSWs was 75 United States Dollar (USD) (inter quartile range (IQR) = 50, 120). Half, 174 (50.3%), of the respondents were grew up in urban areas. With regard to why they left their place of birth, searching for better life was the main reason mentioned by 167 (57.3%) of the respondents (Table 1).

### Reproductive health characteristics

Almost three fourth, (74%, n = 256), of the respondents were not married. The mean ages at first sexual intercourse and marriage were 15.7 years (SD ± 2.3) and 16.2 years (SD ± 2.0), respectively. One hundred ninety three (55.8%) of the respondents had ever give live birth. For those who gave birth, the mean age at first birth was 18.7 years (SD ± 2.8) (Table 2).

**Table 2:** **Reproductive health related characteristics of female sex workers in Mekelle city, northern Ethiopia, 2014 (n = 346)**

**Characteristics**	**Frequency (n)**	**Percent (%)**
**Ever married**		
Yes	90	26.0
No	256	74.0
**Age at first marriage in years (n = 90)**		
10–14	22	24.4
15–25	68	75.6
**Is it willing age for marriage? (n = 90)**		
Yes	54	60.0
No	36	40.0
**Age at first sex in years**		
10–14	55	15.9
15–25	291	84.1
**Ever gave birth before two years**		
Yes	193	55.8
No	153	44.2
**Age at first birth years (n = 193)**		
14–19	130	67.9
20–25	63	32.1
**Total number of pregnancy (n = 234)**		
1–2	190	81.2
>2	44	88.8
**Ever had abortion before 2 years**		
Yes	94	27.2
No	252	72.8
**Number of abortion before 2 years (n = 94)**		
1	77	81.9
>2	17	18.1
**Pregnancy within the past 2 years**		
Yes	115	33.2
No	237	66.8
**Unintended pregnancy within the past 2 years**		
Yes	99	28.6
No	237	71.4
**Type of unintended pregnancy (n = 99)**		
Unwanted	28	28.3
Unplanned	71	71.7
**Reason for unintended pregnancy (n = 99)**		
Condom breakage, slippage, inconsistent/incorrect condom use, forced sexual abuse	38	38.4
Didn’t use condom with steady partner	61	61.6
**Outcome of unintended pregnancy (n = 99)**		
Birth	34	34.3
Abortion	59	59.6
Currently pregnant	5	5.1
Stillbirth	1	1.0
**Currently pregnant**		
Yes	9	2.6
No	337	97.4
**Unintended pregnancy out of the current ones (n = 9)**		
Yes	5	55.6
No	4	45.4

Among the respondents who gave birth, 190 (54.9%) reported having one or two pregnancies and almost half of them 172 (49.7%) had one or two live children. Greater than a quarter, 94 (27.2%), of the respondents had history of abortion before two years and 17 (18.1%) of them reported to experience a single incidence of abortion. In two years prior to the survey period, one third (33.2%) of the respondents reported to have pregnancy, of which 99 (28.6%) were unintended pregnancy. Failure to use condom with steady partner was the major reason for almost two third, 61 (61.6%), of the respondents to have unintended pregnancy. In the two years, 59 women had abortion which accounts to 59.6% of those who had unintended pregnancies and 17.1% of all FSWs. During the survey period, 9 (2.6%) of the respondents were pregnant (Table 2).

### Behavioral related characteristics

The median duration of the respondents in sex work was 24 months (IQR = 24, 42). Almost all, 342 (98.8%), of the respondents knew how to use condom correctly and 326 (94.2%) used condom consistently in the past with their non-regular sexual partners. However, 156 (45.1%) of the respondents reported having history of condom breakage (Table 3).

**Table 3:** **Behavioral related characteristics of female sex workers in Mekelle city, northern, Ethiopia 2014 (n = 346)**

**Characteristics**	**Frequency (n)**	**Percent (%)**
**Do you know how to you use condom correctly?**		
Yes	342	98.8
No	4	1.2
**Did you use condom in the past two years consistently?**		
Yes	326	94.2
No	20	5.8
**Reason for not using condom consistently (n = 20)**		
Clients are not happy	11	55.0
Clients pressure to pay more money,		
not knowing benefits of condom use	9	45.0
**Ever face condom breakage**		
Yes	156	45.1
No	190	54.9
**Measurement taken after condom breakage (n = 156)**		
Tested for HIV at confidential STI clinic	42	26.9
Seek medical advice at health facility	31	19.9
Vaginal douche with soap	65	41.7
Get secret and kept quiet, others*	18	11.5
**Had steady partner**		
Yes	116	33.5
No	230	66.5
**Why do you wish to have steady partner?( n = 116)**		
For support me by money	100	86.2
To prevent me from physical violence	16	13.8
**Do you use condom with your steady partner? (n = 116)**		
Yes	76	65.5
No	40	34.5
**How often do you use condom with your steady partner? (n = 76)**		
Always	25	32.9
Sometimes	51	67.1
**Average sex practice episodes per day**		
1–5	125	36.1
6–10	138	39.9
11–20	83	24.0
**Duration in sex work in months**		
<24	218	63.0
24–48 months	64	18.5
>48 months	64	18.5
**Do you drink alcohol?**		
Yes	223	64.5
No	123	35.5
**Do you use drug?**		
Yes	82	23.7
No	264	76.3
**Have you ever been sexually abused?**		
Yes	19	5.5
No	327	94.5

*Others, wish by urine, wish by lemon.

One third, 116 (33.5%), of the respondents had regular partner. Of these respondents, 40 (34.5%) of them didn’t use condom with their partners. More than two-third, 223 (64.5), of the respondents drank alcohol daily. Almost a quarter, 82 (23.7%), of respondents ever used drugs in the past two years while they were in sex work (Table 3).

### Knowledge and practice on emergency contraceptive

The proportion of the respondents who ever used modern contraceptives are 315 (91%). Almost half, 183 (52.9%), of the respondents never heard about emergency contraceptive methods. Of 163 respondents who heard about emergency contraceptive, only 41 (25.5%) of them reported to ever use the method. Of respondents who had awareness on emergency contraceptive methods, 100 (61.6%) of them reported that the methods can be taken within 120 hours after unsafe sex to prevent unintended pregnancy (Table 4).

**Table 4:** **Knowledge and practices of contraceptive methods among female sex workers in Mekelle city, northern Ethiopia, 2014 (n = 346)**

**Characteristics**	**Frequency (n)**	**Percent (%)**
**Ever contraceptive used**		
Yes	315	91.0
No	31	9.0
**Ever heard/know emergency contraceptive**		
Yes	163	47.1
No	183	52.9
**Emergency contraceptive ever used (n = 163)**		
Yes	41	25.5
No	122	74.5
**When can someone take emergency contraceptive after unprotected sex to prevent pregnancy? (n = 163)**		
Within 120 hours (5 days)	100	61.4
At any time even after 5 days	63	38.6

### Factors associated with unintended pregnancy

In bivariate analysis, factors found to be significantly associated with unintended pregnancy were place of birth, ever gave birth, history of previous abortion, condom utilization, having steady partner, drug use, awareness on emergency contraceptives, duration in sex work and condom breakage. In the multivariate analysis, drug use, having birth, having previous abortion, presence of steady partner and duration in sex work were found to be independent predictors of unintended pregnancy (Table 5).

**Table 5:** **Factors associated with unintended pregnancy among female sex workers in Mekelle city, northern Ethiopia, 2014 (n = 346)**

**Characteristics**	**Unintended pregnancy**		**COR (95% CI)**	**AOR (95% CI)**
	**Yes**	**No**		
**Place of birth**				
Urban	60 (34.5%)	114 (65.5%)	79 (1.11, 2.88)	1.13 (0.59, 2.24)
Rural	39 (22.7%)	133 (77.3%)	1.00	1.00
**Ever gave birth before unintended pregnancy happened**				
Yes	64 (33.3%)	128 (66.7%)	1.70 (1.05, 2.75)	3.07 (1.54, 6.09)***
No	35 (22.7%)	119 (77.3%)	1.00	1.00
**Ever abort before unintended pregnancy happened**				
Yes	63 (67.0%)	31 (33.0%)	12.19 (6.99, 21.26)	15.64 (8.03, 30.47)***
No	36 (14.3%)	216 (85.7%)	1.00	1.00
**Condom use consistently**				
Yes	89 (27.3%)	237 (72.7%)	0.37 (0.15, 0.93)	0.72 (0.21, 2.43)
No	10 (10.1%)	10 (4%)	1.00	1.00
**Had steady partner**				
Yes	45 (38.8%)	71 (61.2)	2.06 (1.27, 3.34)	2.87 (1.47, 5.61)**
No	54 (23.5%)	176 (76.5)	1.00	1.00
**Drug user**				
Yes	39 (47.6%)	43 (52.4%)	3.08 (1.83, 5.18)	2.68 (1.30, 5.52)**
No	60 (22.7%)	204 (77.3%)	1.00	1.00
**Ever heard emergency contraceptives**				
Yes	58 (35.6)	105 (64.4%)	1.91 (1.19, 3.07)	1.29 (0.70, 2.39)
No	41 (22.4%)	142 (77.6%)	1.00	1.00
**Duration of sex work**				
<12 months	36 (22.8%)	122 (77.2%)	1.00	1.00
12–24 months	23 (38.3%)	37 (61.7%)	2.10 (1.11, 3.99)	1.51 (0.67, 3.40)
25–59 months	21 (32.8%)	43 (67.2%)	1.65 (0.87, 3.14)	0.78 (0.33, 1.79)
60–96 months	12 (22.6%)	41 (77.4%)	0.99 (0.47, 2.08)	0.33 (0.11, 0.95)*
>96 months	7 (63.6%)	4 (36.4%)	5.93 (1.64, 21.40)	1.81 (0.33, 9.76)
**Condom breakage**				
Yes	52 (33.3%)	104 (66.7%)	1.52 (0.95, 2.43)	1.04 (0.57, 1.91)
No	47 (24.7%)	143 (75.3%)	1.00	1.00

*P <0.05; **P < 0.01; ***P < 0.001; AOR-Adjusted Odds Ratio; COR-Crude Odds Ratio; CI-Confidence Interval.

FSWs who ever gave birth had 3.1 (AOR = 3.07, 95% CI: [1.54, 6.09]) times more likely to have unintended pregnancy compared to those who do not. FSWs who ever had abortion were 15.6 (AOR = 15.64 95% CI: [8.03, 30.47]) times more likely of having unintended pregnancy than who never had. FSWs who had steady partner had 3 (AOR = 2.87, 95% CI: [1.47, 5.61]) times more likely to have unintended pregnancy compared to their counter parts. FSWs who were drug users had 2.7 (AOR = 2.68, 95% CI: [1.30, 5.52]) times more likely to have unintended pregnancy than those who didn’t use. FSWs whose duration of sex work was in the interval of 60–96 months were 67% less likely to have unintended pregnancy than those with <12 months duration of sex work (AOR = 0.33, 95% CI: [0.11, 0.95]) (Table 5).

## Discussion

In the current study, 99 (28.6%) respondents had unintended pregnancy; of which three-fifths, 59 (59.6%), of unintended pregnancies ended with abortion. The prevalence of unintended pregnancy in this study was higher than a study done in Ethiopia from the general population (24%) [8]. This could be explained by the fact that FSWs are at higher risk of unintended pregnancy than the general population [4,5]. Studies done in the pocket areas of Ethiopia revealed that unintended pregnancy rate ranges from 27.9% to 42.4% [10,11]. In contrast to our findings, studies conducted among FSWs in Afghanistan, Swaziland and Kenya showed much higher prevalence of unintended pregnancy with 36.9%, 49% and 52%, respectively [6,15-17]. This could be due to the fact that the efforts of confidential STI and FGAE clinics in study area might have contributed better coverage of contraceptive utilization among FSWs compared to the other SSA countries [6,17].

Unintended pregnancy and unsafe abortion as a result of low contraceptive utilization are prevalent in Ethiopia (*Mamboleo N: Unwanted pregnancy and induced abortion among female youths: a case study of Temeke district, Dar es Salaam, Tanzania, unpublished*. [12]) and they are the main causes of maternal mortality. Ethiopia has the fifth highest number of maternal deaths in the world—one in 27 women die from complications of pregnancy or child birth annually [7-11]. In sub-Saharan Africa, women who are engaged in commercial sex are at higher risk of physical and sexual violence, unintended pregnancy and STIs including HIV/AIDS [6,18].

Ever having previous history of birth and abortion was significantly associated with unintended pregnancy in this study. The finding is consistent with results of other studies conducted in Afghanistan, Savannakhet and Moscow [15,19,20]. Having a steady partner was a major risk factor associated with unintended pregnancy. Similar to our study, a research from Swaziland reported that FSWs who have non-commercial and often more intimate partners are less likely than others to be protected against STIs and unintended pregnancy [16]. The fact that FSWs expect to obtain a support from their steady partners if any consequences of unprotected sex (like unintended pregnancy and STI) occur might be the reason. The other reason might be also FSWs could have better understanding and trust in their steady partners than casual sex customers to have unprotected sex.

Being drug users was also another significant factor associated with unintended pregnancy. This finding is in line with results of studies conducted in Afghanistan, which revealed that drug use and duration of sex work was significantly associated with unintended pregnancy [15,21]. Studies suggested that drug addicted FSWs are more likely to be exposed for unprotected sexual intercourse as well as for higher risk of unintended pregnancy [4,5].

Mentioning limitations of this study will be worthy. The major limitation of this study emanated from the nature of the issue which is sensitive and may introduce social desirability bias which in turn underestimates the magnitude of the problem in this specific group. Since the research is cross sectional, the causality inference of the study variables might be limited. Besides, recall bias and miss reporting of events is likely to happen. Consideration of qualitative study would have been important to look on further reasons and factors associated with unintended pregnancy. Furthermore, in the current study, we have considered individual level factors only. Investigations on health service related factors would have been important in the study. In addition, the study didn’t categorically differentiate types of abortions among FSWs.

## Conclusions

In conclusion, significant magnitude of unintended pregnancy was found among FSWs. In the current study, abortion and drug use were also prevalent among FSWs. Previous history of birth, previous history of abortion, having steady sexual partner and drug use were positively associated with unintended pregnancy. Unintended pregnancy associated consequences could lead to poor reproductive and general health of FSWs. Unintended pregnancies could be also associated with increased risk of STI/HIV transmission among FSWs and in the general community.

High level of unintended pregnancy and abortion as a result of unsafe sex (particularly due to underutilization of effective contraceptive methods) suggests that FSWs may not have access, detail knowledge or resources for reproductive health services. Hence, access in terms of increasing availability and accessibility of contraceptives as well as provision of compassionate care to FSWs is crucial to reduce unintended pregnancy in this group. Ongoing and continuous counseling on safe sex, including correct and consistent use of condom and, for particular clients, enhancing use of emergency contraceptive methods will benefit to reduce unintended pregnancy among FSWs. As this population group is special and difficult to reach with reproductive health interventions, tailored strategies and mechanisms should be developed to address unintended pregnancy and its consequence.
